# Transgenic Bt rice lines producing Cry1Ac, Cry2Aa or Cry1Ca have no detrimental effects on Brown Planthopper and Pond Wolf Spider

**DOI:** 10.1038/s41598-017-02207-z

**Published:** 2017-05-16

**Authors:** Lin Niu, Amani Mannakkara, Lin Qiu, Xiaoping Wang, Hongxia Hua, Chaoliang Lei, Juan Luis Jurat-Fuentes, Weihua Ma

**Affiliations:** 1 Hubei Insect Resources Utilization and Sustainable Pest Management Key Laboratory, College of Plant Science and Technology, Huazhong Agricultural University, Wuhan, 430070 Hubei China; 2 Department of Agricultural Biology, Faculty of Agriculture, University of Ruhuna, Kamburupitiya, 81100 Sri Lanka; 3 Department of Entomology and Plant Pathology, University of Tennessee, Knoxville, TN 37996 USA

## Abstract

Transgenic rice expressing *cry* genes from the bacterium *Bacillus thuringiensis* (Bt rice) is highly resistant to lepidopteran pests. The brown planthopper (BPH, *Nilaparvata lugens*) is the main non-target sap-sucking insect pest of Bt transgenic rice. The pond wolf spider (PWS, *Pardosa pseudoannulata*) is one of the most dominant predators of BPH in rice fields. Consequently, the safety evaluation of Bt rice on BPH and PWS should be conducted before commercialization. In the current study, two experiments were performed to assess the potential ecological effects of Bt rice on BPH and PWS: (1) a tritrophic experiment to evaluate the transmission of Cry1Ac, Cry2Aa and Cry1Ca protein in the food chain; and (2) binding assays of Cry1Ac, Cry2Aa and Cry1Ca to midgut brush border membrane proteins from BPH and PWS. Trace amounts of the three Cry proteins were detected in BPH feeding on Bt rice cultivars, but only Cry1Ac and Cry2Aa proteins could be transferred to PWS through feeding on BPH. *In vitro* binding of biotinylated Cry proteins and competition assays in midgut protein vesicles showed weak binding, and ligand blot analysis confirmed the binding specificity. Thus, we inferred that the tested Bt rice varieties have negligible effects on BPH and PWS.

## Introduction

Rice (*Oryza sativa*) is one of the most important food crops in Asia^1^. There are more than 200 species of insect pests that infect rice during its growing season^2, 3^, including the striped stem borer (*Chilo suppressalis*) and leaf-folders (*Cnaphalocrocis medinalis*), which are chronic lepidopteran pests responsible for large annual losses^4, 5^. Traditional management of lepidopteran pests relies on chemical pesticides that not only cause environmental contamination and potential risks to human health, but may also reduce the populations of beneficial predatory insects^6, 7^. Transgenic plants containing *cry* genes have been proven effective against lepidopteran insect pests^8^ and have been successfully developed for the management of caterpillars^3, 9–13^. However, similar to other plant protection strategies, the adoption of Bt rice may have potential risks to the environment. One of the main concerns of using transgenic Bt crops is their potential impact on non-target herbivorous insects and their predators, which provide important ecological functions^14–16^.

The brown planthopper (BPH, *Nilaparvata lugens* Stål) (Hemiptera: Delphacidae) is found in most rice fields worldwide^17, 18^. This insect is easy to rear in the laboratory, which makes it an ideal arthropod candidate for the evaluation of potential risks associated with transgenic rice. Former publications on the effects of Bt rice on BPH address the survival, growth, oviposition behavior^19–23^, or field population dynamics of the insect^11, 23–25^. Our previous study has showed that Bt rice has no detrimental effects on the digestion, detoxification and immune responses of BPH^22^. In addition, Bt toxin was detected in the honeydew of BPH after being fed on transgenic Bt rice, but no effects on the fitness of the BPH or its predator *Cyrtorhinus lividipennis* were observed^26^. The Cry1Ab protein maybe transferred from transgenic rice plants to BPH^27^, and from BPH to its predator wolf spider (WS, *Pirata subpiraticus*)^28^. However, the possibility of insecticidal proteins from Bt rice binding to the BPH midgut remains unclear.

Spiders are generalist predators and prey on insect pests that infect crops^29, 30^. The Cry proteins produced by Bt rice may be transferred to the predators through the herbivorous insects feeding on Bt rice plants^28^. The pond wolf spider (PWS, *Pardosa pseudoannulata*) is one of the dominant spider species in Chinese farmlands^31^. Tian *et al*.^32^ demonstrated that Cry1Ab toxin could be transferred from the Bt rice lines KMD1 and KMD2 through the BPH to PWS, but that the tested Cry1Ab rice line did not influence the spider’s fitness^32^. Zhou *et al*.^31^ showed that the activities of three key metabolic enzymes were significantly influenced in PWS after feeding on Cry1Ab-containing fruit flies^31^. Moreover, Bernal *et al*.^26^ detected Cry1Ab toxin in honeydew from BPH and concluded that BPH and its predator *C*. *lividipennis*, could be exposed to Bt toxins from Bt rice^26^. Cry1Ab from Bt rice can be transferred to BPH and thus expose its predator *Propylea japonica* ^27^, but no adverse effects have been found on any of the fitness parameters. When supplied with Bt rice-fed BPH, the Cry1Ab protein was detected in *Ummeliata insecticeps*, but no effects on its survival and development were observed^33^. Despite these previous reports, very few studies have detected the binding of the Cry protein in the predator spider^34^, and the Cry binding protein in PWS is still unknown.

In the current study, we used three transgenic Bt rice lines producing Cry1Ab/1Ac fused proteins, Cry2Aa or Cry1Ca proteins to investigate the effects of Bt toxins on the non-target insect BPH and its predator PWS. The work reported here had 2 objectives: (1) to quantify the Cry proteins in both BPH after being fed on Bt rice and in PWS after feeding on BPH that had been reared on Bt rice; (2) to evaluate the binding of the three Cry proteins (Cry1Ac, Cry2Aa and Cry1Ca) produced by the rice lines in midgut brush border membrane vesicles (BBMVs) from BPH and PWS.

## Results

### Cry1Ac, Cry2Aa and Cry1Ca protein detection in BPH and PWS

Detection by ELISA assays showed that Cry1Ac, Cry2Aa and Cry1Ca proteins could be detected in BPH after feeding on the three tested Bt rice plant lines, and the toxin concentration was 3.6 ± 1.7 ng/g, 9.8 ± 2.7 ng/g and 1.7 ± 0.8 ng/g of fresh weight, respectively (Table 1). No Cry proteins were detected in the BPH reared on non-transgenic rice isoline (MH 63).

**Table 1:** Detection of Cry1Ac, Cry2Aa and Cry1Ca proteins in BPH after feeding on Bt rice and and PWS after predating on the BPH.

Transgene protein	Treatments	Contents of transgene proteins ± SE (ng/g fresh weight)
Cry1Ac	BPH provided with TT51	3.6 ± 1.7
	PWS provided with TT51 BPH	2.5 ± 0.9
	BPH provided with MH63	Not detectable
	PWS provided with MH63 BPH	Not detectable
Cry2Aa	BPH provided with T2A-1	9.8 ± 2.7
	PWS provided with T2A-1 BPH	6.5 ± 3.9
	BPH provided with MH63	Not detectable
	PWS provided with MH63 BPH	Not detectable
Cry1Ca	BPH provided with T1C-19	1.7 ± 0.8
	PWS provided with T1C-19 BPH	Not detectable
	BPH provided with MH63	Not detectable
	PWS provided with MH63 BPH	Not detectable

Both the Cry1Ac and Cry2Aa proteins could be transferred from BPH to PWS by predation, while Cry1Ca protein could not. The concentration of Cry1Ac and Cry2Aa in PWS adults was 2.5 ± 0.9 ng/g and 6.5 ± 3.9 ng/g of fresh weight when predating on BPH that were fed TT51 and T2A-1 rice, respectively (Table 1). No Cry proteins were detected in the PWS predating on BPH fed the non-transgenic rice isoline (MH 63).

### Binding of Cry1Ac, Cry2Aa and Cry1Ca to BPH and PWS BBMV

**Figure 1:**
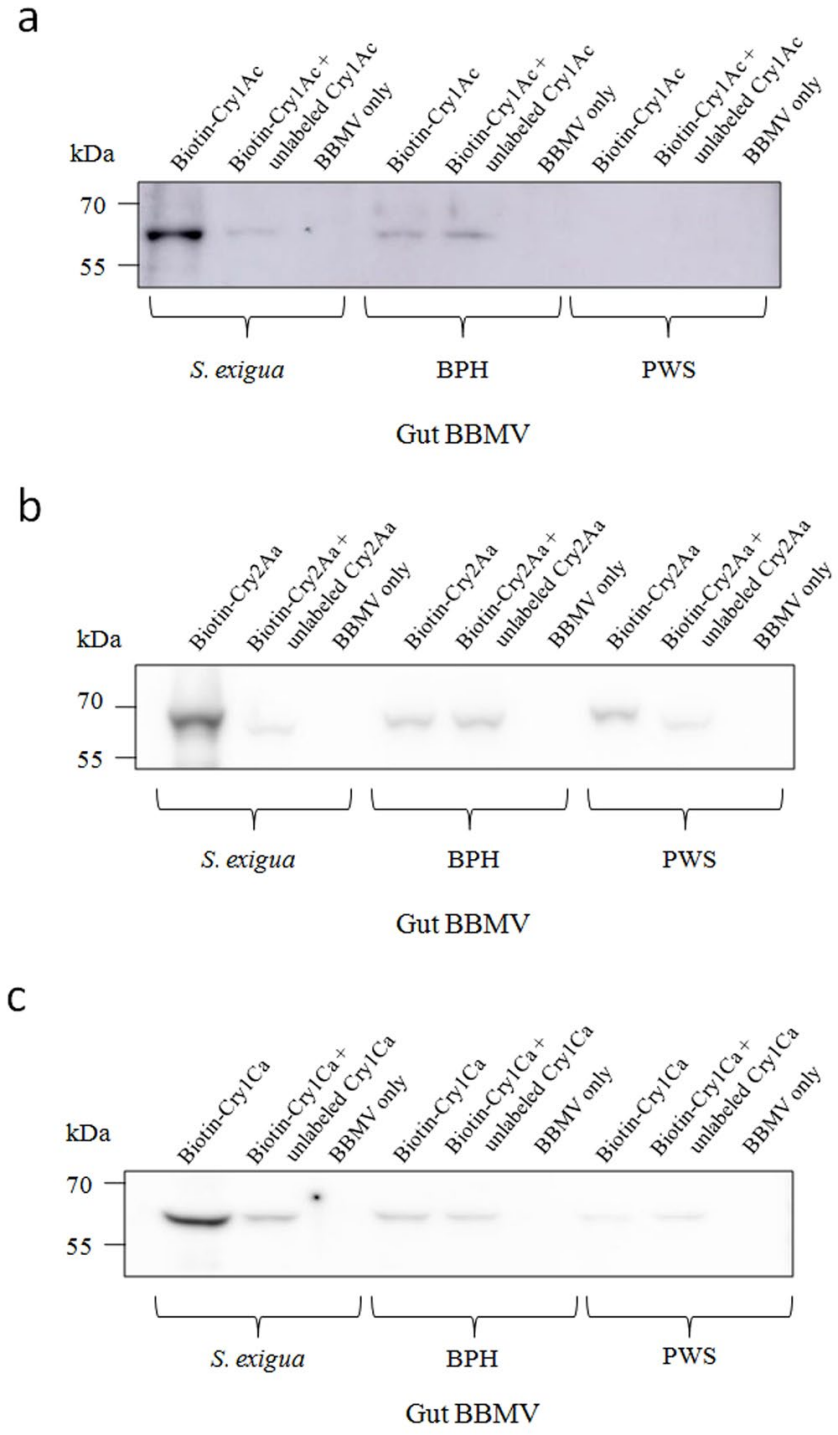
Binding of biotinylated Cry1Ac (**a**), Cry2Aa (**b**) and Cry1Ca (**c**) to BPH and PWS gut BBMV. Twenty micrograms of BBMV protein were used along with 0.1 micrograms of biotinylated Cry proteins. A 100-fold excess of unlabeled Cry1Ac, Cry2Aa or Cry1Ca was used in competition assays. Toxin binding to *S*. *exigua* gut BBMV was used as a positive control treatment.

Binding assays were conducted with biotinylated Cry proteins to investigate the binding of Cry1Ac, Cry2Aa and Cry1Ca to midgut proteins of BPH and PWS. In these assays, we used midgut brush border membrane vesicles from *Spodoptera exigua* as a positive binding control based on its susceptibility to Bt cotton producing Cry1Ac and Cry2Ab^35^ and to Cry1Ca^36^ toxin, and previous reports demonstrating high affinity specific binding of the toxins to *S*. *exigua* BBMV^37, 38^. There was no evidence for specific binding of any of the three tested proteins to BBMV from BPH and PWS (Fig. 1a–c). For *S*. *exigua* BBMV, binding of biotinylated-Cry1Ac, biotinylated-Cry2Aa and biotinylated-Cry1Ca toxin was easily detected and displaced by 100-fold excess of unlabeled homologous competitor proteins (Fig. 1a–c), supporting specific binding. In contrast, 100-fold excess of unlabeled Cry proteins did not reduce the weak binding of biotinylated Cry proteins to BBMV from BPH and PWS, supporting that the three Cry proteins tested had no specific binding to these BBMV.

**Figure 2:**
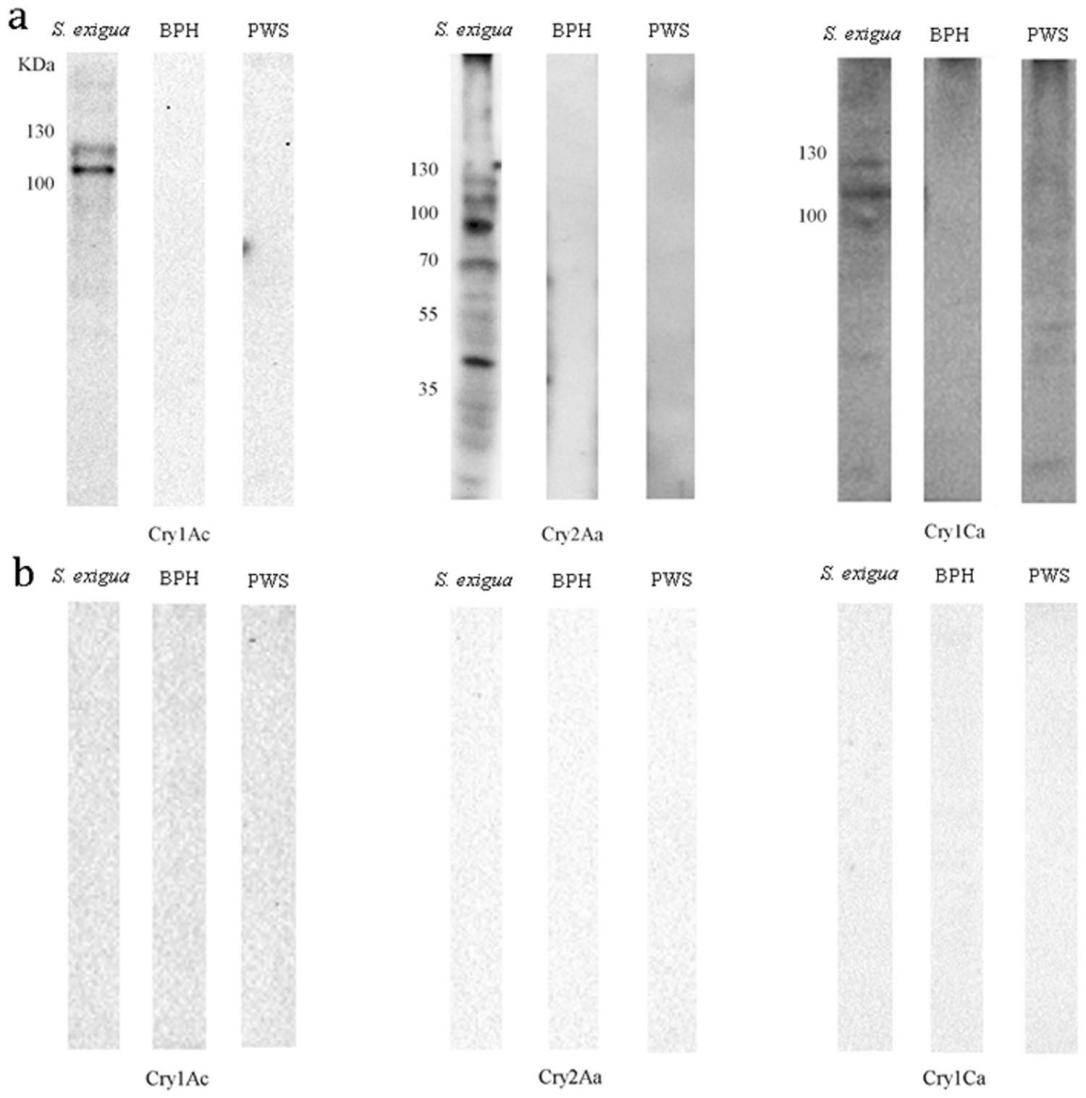
Ligand blots of Cry1Ac, Cry2Aa and Cry1Ca toxins with BPH and PWS BBMV proteins. (**a**) PVDF membranes from BPH and PWS BBMV were incubated with activated Cry toxin, primary antibody, and secondary antibody. (**b**) PVDF membranes from BPH and PWS BBMV were incubated with primary antibody and secondary antibody. *S*. *exigua* gut BBMV proteins were used as a positive control treatment.

To further test interactions between midgut proteins of BPH and PWS with Cry proteins, we performed ligand blotting analyses. Our results showed that in *S*. *exigua* BBMV Cry1Ac recognized proteins of approximately 110-, 120-kDa in size (Fig. 2a), Cry2Aa bound to proteins of approximately 40-, 70-, 90-, 110- and 120-kDa (Fig. 2a), and Cry1Ca mostly recognized two proteins of approximately 110- and 120-kDa (Fig. 2a). In contrast, we did not observed distinct binding of Cry1Ac, Cry2Aa or Cry1Ca to the BBMV proteins from BPH and PWS (Fig. 2a). Negative controls including BBMV proteins without exposure to Cry toxins (Fig. 2b) did not detect any binding interactions.

## Discussion

Non-target risk assessment for transgenic crops should be case specific, and consider variables including the plant, transgene and environment^39^. In the present study, we focused on the potential ecological risk of transgenic Cry1Ac, Cry2Aa and Cry1Ca rice to BPH and its predator PWS at the molecular level. We detected the transmission of Cry proteins in the Bt rice-BPH-PWS food chain, and performed binding and ligand blotting assays testing the binding of Cry toxins to BPH and PWS BBMV. This is the first reported study to evaluate Cry toxin binding in BPH and PWS.

Transmission of the Cry1Ac, Cry2Aa and Cry1Ca proteins from Bt rice to BPH and PWS was quantified by ELISA (Table 1). Low levels of the tested Cry toxins could be detected in BPH after feeding on Bt rice (less than 10 ng/g fresh weight). Cry1Ac and Cry2Aa toxin could be further transferred to PWS during predation on BPH fed on Bt-rice. However, we found Cry1Ca could not be transferred. These results are in agreement with previous reports examining whether Bt proteins can be transferred to predators from BPH feeding on transgenic Bt rice. For example, Cry1Ab could be transferred to *P*. *subpiraticus* through Bt rice-BPH-predator food chain, and the Cry1Ab protein in *P*. *subpiraticus* was significantly higher than that in BPH fed on Bt rice^28^. Cry1Ab was also transferred to *U*. *insecticeps* through the food chain, but the concentration of Cry1Ab in *U*. *insecticeps* was much lower than that in BPH^33^. In addition, Cry1Ab and Cry2A proteins were transferred to *C*. *lividipennis* and *Hylyphantes graminicola* via predation on BPH fed on transgenic Bt rice^40, 41^. However, and in contrast to our observations, Han *et al*. (2014) concluded that Cry2A protein concentration was very low in BPH and could not be transferred to *C*. *sinica* and *C*. *lividipennis* by preying on BPH^16^. In agreement with our findings, Meng *et al*. found that the concentration of Cry1C in BPH fed with T1C-19 was 1.1 ± 0.0 ng/g, and could not be transferred to *P*. *fuscipes* via predation^42^. The differences in detection results may be attributed to the different exposure times and/or degradation of Bt toxin. For instance, Zhao *et al*.^43^ found that the longer ladybeetles consumed aphids that were feeding on Bt plants, the more toxin it accumulated in the predator’s body^43^. Tian *et al*.^44^ also found that Cry1Ab protein could accumulate in the spider via the Bt rice-BPHs-PWS food chain, despite the degradation of the Cry1Ab protein^44^.

The action of Bt Cry toxins includes a critical binding step to receptors in the insect midgut^45^. Interactions between Cry toxins and midgut proteins of non-target insects would support that the possibility of detrimental effects of Bt crops producing those toxins exist. Previous studies did find low concentrations of Cry toxin residues in the midguts of BPH upon feeding on Bt rice that did not affect survival and growth of the BPH^16, 42, 46^. However, the potential interaction between Cry toxins and midgut proteins of BPH and PWS was not tested. Rodrigo-Simón *et al*.^47^ showed that Cry1A proteins failed to bind to green lacewing (*Chrysoperla carnea*) BBMV and suggested that this explained lack of adverse effects^47^. Ferry *et al*.^48^ did not find a Cry3A binding protein in *Nebria brevicollis* BBMV and suggested that this explained the lack of acute or chronic effects of Cry3A on adults of *N*. *brevicollis* ^48^. However, Li *et al*.^49^ found that Cry1Ac could bind to the aphid gut epithelium, yet only low aphid toxicity was detected in bioassays^49^. In our present study, none of the tested Cry proteins displayed specific binding to BBMV from BPH or PWS. Results from ligand blotting experiments provided further support for the lack of specific binding sites for the toxins in BPH or PWS midgut. Consequently, even considering potential transmission of the tested three Bt toxins to BPH and its predator PWS, no adverse effects are expected.

In summary, the current study supports that Bt rice lines TT51, T2A-1 and T1C-19 have no adverse effects on BPH or its predator PWS. Since these three Bt rice plants have not been promoted in China, the present study can provide information for the commercialization of new Bt varieties for agricultural protection.

## Materials and Methods

### Test plant materials

Three Bt rice strains (TT51, T2A-1 and T1C-19) generated by the Huazhong Agriculture University and their non-transgenic parental indica cultivar Minghui 63 (MH 63) were used in this study. TT51 expresses a Bt fusion gene derived from Cry1Ab and Cry1Ac under the control of rice actinI promoter^50^. T2A-1 expresses one synthesized Cry2Aa gene^10^ and T1C-19 expresses one synthesized Cry1Ca gene^12^, expression of both of them was driven by the maize ubiquitin promoter. MH 63 served as the non-transgenic control isoline. The three transgenic Bt rice strains exhibit high resistance against lepidopteran pests^10, 12, 50^.

### BPH and PWS preparation

Adults of BPH were randomly collected from paddy fields in Wuhan, Hubei Province, China. BPHs were exposed to the 3 tested Bt rice or control rice lines on 15-day-old rice seedlings cultured with Yoshida solution in glass bottles, and used as spider diets as described below.

The PWS larvae were obtained from the eggs of a single female collected from the experimental farmland in Huazhong Agricultural University, Wuhan, Hubei Province, China, and reared in a glass tube (12 mm × 100 mm) with BPHs. Each spider larva was individually placed in a tube with a moist cotton ball to provide enough water for its survival and supplied with 20 BPHs daily. The majority of spiders preyed on 12 BPHs per day^44^.

### Quantification of Bt toxin in BPH and PWS

BPHs were segregated into four groups, three fed with transgenic rice and one with normal rice. After feeding for 15 days, the BPHs were fed to PWS. Nymphs of BPH (30 per treatment) and PWS adults (10 per treatment) were collected for detection of Cry protein. Levels of Cry toxin accumulation in BPHs and PWSs were measured with an enzyme-linked immunosorbent assay (ELISA) using the EnvirologixQualiplate Kit (EnviroLogix Inc., Portland, Me, USA). Before the assay, the samples were washed with PBST buffer (PBS/0.5% Tween-20) to remove the Cry protein from their surfaces. Then BPHs and PWSs were homogenized in the extract buffer (provided by the kit) and centrifuged for 10 min at 13,000 × *g*. The supernatants were used for ELISA analyses.

### Binding and competition assays using isolated BPH and PWS BBMVs

Midguts of BPH were dissected from macropterous female adults (soon after ecdysis) to prepare BBMV using methods described previously^51, 52^. For preparation of BBMV from PWS we used dissected gut tissue from 7-day old adult spiders. Briefly, more than 1,000 BPH nymphs and 20 PWS adult gut tissues were collected in 1.5 ml MET buffer (0.3 M Mannitol, 5 mM EGTA, 17 mM Tris-HCl pH 7.5) containing protease inhibitors (PMSF) to prevent protein degradation, and stored at −80 °C until used. The isolated guts were homogenized and the extracted BBMV pellets resuspended in ice-cold MET buffer with protease inhibitors, and then flash frozen with liquid nitrogen and stored at −80 °C until used. Preparation of *S*. *exigua* BBMV was by the differential centrifugation method of Wolfersberger^51^. The protein concentration of the BBMV was determined by the Bradford method (BSA was used as the standard protein) according to the manufacturer’s instructions.

Toxin labeling and binding of biotinylated toxins were carried out as described elsewhere^53^. Active Cry1Ac, Cry2Aa and Cry1Ca toxins (1 mg) were labeled with biotin by incubation with 10 nM EZ-Link NHS-LC-Biotin (Thermo Scientific) in PBS buffer at room temperature for 1 h. Free biotin was removed by dialysis overnight in 4 liters of 20 mM Na_2_CO_3_, 150 mM NaCl at 4 °C. Protein concentration of biotinylated Cry1Ac, Cry2Aa and Cry1Ca toxins was determined with the Qubit Protein Assay kit (Invitrogen) following the manufacturer’s instructions, and then proteins were stored in aliquots at −80 °C.

Binding reactions (100 μl final volume) included 20 μg of *S*. *exigua*, BPH or PWS BBMV proteins and 0.1 μg of biotinylated Cry1Ac, Cry2Aa or Cry1Ca in binding buffer (PBS plus 0.1% BSA), and were allowed to proceed for 1 h at room temperature. Reactions were stopped by centrifugation for 10 min at 15,000 × *g* at 4 °C, and then BBMV and bound toxin in pellets were washed with 0.5 ml of ice-cold binding buffer, and these steps were repeated for a total of three times. Final pellets were solubilized in 10 μl of SDS sample buffer, and heat-denatured at 100 °C for 5 min. The samples were resolved by 10% SDS-PAGE and electrotransferred to polyvinylidene difluoride (PVDF) membranes. After blocking blots for 1 h at room temperature in PBS buffer (135 mM NaCl, 2 mM KCl, 10 mM Na_2_HPO_4_, 1.7 mM KH_2_PO_4_, pH 7.5) containing 3% bovine serum albumin (BSA) and 0.1% Tween 20, filters were probed with streptavidin-HRP conjugate (1:20,000) for 1 h at room temperature. Membranes were washed with washing buffer (PBS plus 0.1% BSA and 0.1% Tween 20) for 1 h (10 min per wash). After the last wash, the bound toxins were visualized using the ECL chemiluminescence detection kit (Thermo Fisher Scientific, Waltham, MA USA). For competition assays, the same protocol was followed except that a 100-fold excess of unlabeled homologous toxin was included in the binding reactions.

Ligand blot analyses were performed using 20 μg of *S*. *exigua*, BPH or PWS BBMV proteins resolved by 8% SDS-PAGE and then transferred to polyvinylidene difluoride (PVDF) filters. Filters were blocked for 2 h at room temperature with PBST buffer (PBS buffer plus 0.1% Tween-20) containing 5% (w/v) nonfat dry milk powder. Subsequently, PVDF filters were separately incubated with 2 μg/ml of biotinylated Cry1Ac, Cry2Aa or Cry1Ca in PBST buffer containing 5% of milk powder (blocking buffer) overnight at 4 °C. Filters were then washed three times with PBS buffer containing 0.1% Tween-20 and incubated in blocking buffer for 2 h with polyclonal rabbit anti-Cry1Ac, anti-Cry2Aa (1:3,500) or monoclonal mouse anti-Cry1Ca (1:5,000) sera. After washing, filters were probed with polyclonal HRP-conjugated goat anti-rabbit secondary antisera (for Cry1Ac and Cry2Aa, 1:5,000) or goat anti-mouse secondary antisera (for Cry1Ca, 1:5,000). After washing, filters were developed using the ECL chemiluminescence detection kit (Fermentas/Thermo Fisher Scientific, Waltham, MA USA).
